# A Reverse Localization Scheme for Underwater Acoustic Sensor Networks

**DOI:** 10.3390/s120404352

**Published:** 2012-03-29

**Authors:** Marjan Moradi, Javad Rezazadeh, Abdul Samad Ismail

**Affiliations:** Department of Computer Systems and Communication, Universiti Teknologi Malaysia, UTM Skudai 81310, Johor, Malaysia; E-Mails: rezazadeh@ieee.org (J.R.); abdsamad@utm.my (A.S.I.)

**Keywords:** localization, underwater wireless sensor networks, reverse localization scheme

## Abstract

Underwater Wireless Sensor Networks (UWSNs) provide new opportunities to observe and predict the behavior of aquatic environments. In some applications like target tracking or disaster prevention, sensed data is meaningless without location information. In this paper, we propose a novel 3D centralized, localization scheme for mobile underwater wireless sensor network, named Reverse Localization Scheme or RLS in short. RLS is an event-driven localization method triggered by detector sensors for launching localization process. RLS is suitable for surveillance applications that require very fast reactions to events and could report the location of the occurrence. In this method, mobile sensor nodes report the event toward the surface anchors as soon as they detect it. They do not require waiting to receive location information from anchors. Simulation results confirm that the proposed scheme improves the energy efficiency and reduces significantly localization response time with a proper level of accuracy in terms of mobility model of water currents. Major contributions of this method lie on reducing the numbers of message exchange for localization, saving the energy and decreasing the average localization response time.

## 1. Introduction

During the last decades, there has been a rapidly growing interest in monitoring aqueous environments for scientific exploration, ecosystem monitoring and early warning systems for natural disasters like tsunamis. The ideal means for this type of extensive monitoring is a distributed underwater system with networked wireless sensors, referred to as Underwater Wireless Sensor Network (UWSN) [1]. In some of these applications such as oil drilling, target or animal tracking and disaster prevention the sensed data should be tagged with location information because applications associate the sampled values with when and where they were collected [2].

Localization in terrestrial wireless sensor networks (TWSN) is mature enough, while it is still challenging for UWSN due to some major technical differences. Acoustic communications are the typical physical layer technology in underwater networks. In fact, radio waves propagate at long distances through conductive salty water only at extra-low frequencies (30–300 Hz), which requires large antennae and high transmission power. Optical waves cannot be used because even though they do not suffer from such high attenuation, they are seriously affected by scattering. Furthermore, transmitting optical signals requires high precision in pointing the narrow laser beam. Thus, links in underwater networks are typically based on acoustic wireless communication [3]. Using acoustic communication among UWSN compared to radio links in TWSN presents different challenges and constraints in underwater localization. It is challenging as Radio Frequency (RF) waves are heavily attenuated under water, so employing technology like GPS is not feasible [4]. The acoustic channels are characterized by severely limited bandwidth, high propagation delays and high bit error rates. Hence, it is desired that localization protocols work with minimum possible message exchange. Underwater acoustic communication links can be classified according to their range as *short, medium, long* and *very long*. Data rates of acoustic links and typical bandwidths of the underwater acoustic channel for different ranges are given in Table 1 [5,6].

As shown in Table 1, bandwidth and data rates of acoustic underwater communications is very low, but increase for shorter distances. This implies, in a range of applications with using long range acoustic modems, localization protocols with the least possible messages and low data bits should be established. Otherwise, their performance is drastically affected by protocol overhead [2]. This is also constrained by limited power of the underwater sensors and impossibility of changing or recharging the battery [7]. Meanwhile, underwater sensor networks if not anchored, are mobile networks and node locations change continuously. So, the mobility of free-floating nodes brings up another challenge in localization. In such a dynamic aquatic environment, localization process should be run periodically to update the location results, as will dramatically increase the communication overhead and energy consumption [7,8]. Moreover, in some applications like forewarning disaster and coastline prevention, the localization response time should be fast so that it reports the actual location when data is sensed [9].

In this paper, we present the design and the development of a new message exchange mechanism which can provide an energy efficient localization scheme with the least number of localization applicant messages. The proposed scheme supports the mobility feature of water currents and significantly increases average response time for localization. We propose a fast and energy efficient localization method by transferring the location estimation from sensors to base station. Our scheme aims to minimize the number of localization messages while decreasing the average response time and keeping the accuracy considering to the mobility feature of water currents.

The rest of the paper is organized as follows: Section 2 discusses the research background. The detailed localization scheme is discussed in Section 3. Section 4 discusses the error analysis. Section 5 shows the simulation results. Advantages and disadvantages of the proposed approach are described in Section 6 and we conclude our work in Section 7.

## 2. Research Background

Localization is known as location estimation of ordinary sensor nodes in a network. Most localization schemes need the location of some nodes to be known. These location-aware nodes are known as anchor or beacon nodes [4]. There are different methods to prepare location information for the anchors such as placed at fix location or using special hardware like Global Positioning System (GPS) [10]. A typical localization process comprises the following steps [9]:

- Range measurement
- Location estimation
- Calibration

In terms of range measurement, the localization schemes can be classified into two categories: range-based and range-free. In range-based schemes, precise estimations of distance or angle are made to estimate the location of nodes [11]. Different techniques are available to calculate distances to other nodes like: Time of Arrival (TOA), Time Difference of Arrival (TDOA), Angle of Arrival (AOA) or Received Signal Strength Indicator (RSSI), while range-free localization schemes do not use range or bearing information. In contrast, range-free location estimation methods are based on connectivity information instead of distance or angle measurements [11].

In the second step, for estimating the location of an unknown node, two well-known range-based localization techniques include angulation and lateration utilizing bearing and distance information, respectively [10]. Range-based methods provide fine-grained location estimation. Moreover, in UWSNs, acoustic channels are naturally employed and range measurement using acoustic signals are much more accurate than using radio [12,13]. However, range-free schemes only provide a coarse estimate of a node's location, but the advantage of these schemes lies in their simplicity. Range-free localization techniques are classified into hop count-based and area-based methods [4].

In step three, the estimated location is refined via measurements from various iterations, measurement error models, mobility models, *etc.* [9].

### 2.1. Underwater Localization Survey

**Figure 1.**
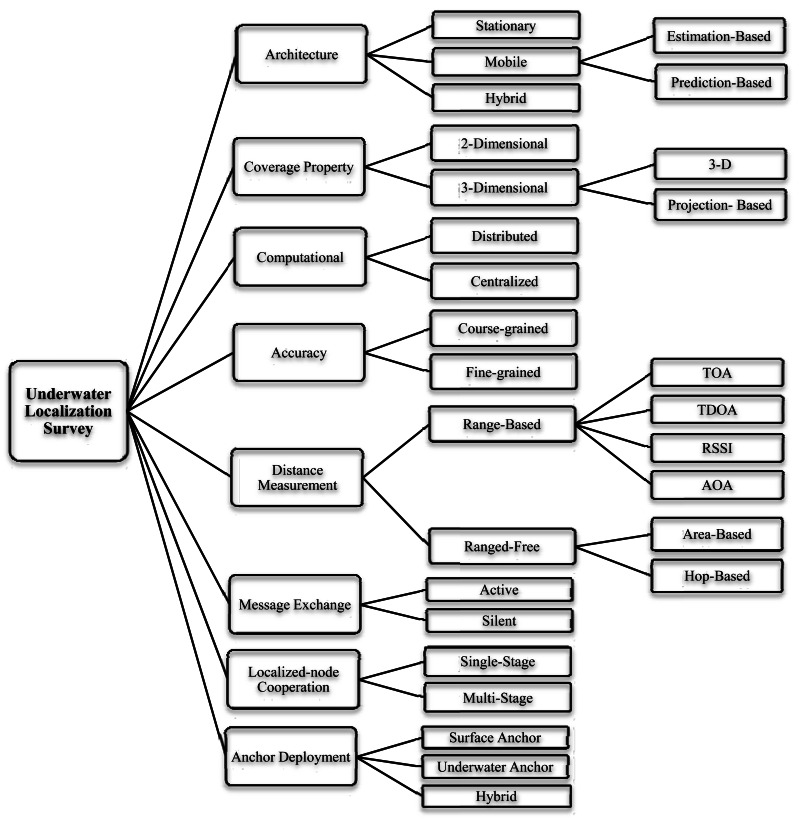
Localization Survey.

In this section, we broadly categorize different types of localization issue based on various properties. Figure 1 illustrates the underwater localization survey.

- *Underwater Sensor Architecture*: Underwater networks comprise various sensors such as: surface buoys, anchor or beacon nodes, ordinary sensor nodes and Autonomous Underwater Vehicles (AUV). They have different motion capabilities like stationary, mobile or hybrid. In stationary case, nodes have fixed locations while mobile nodes are drifted with water currents or their motion can be controlled by navigation devices. In hybrid architectures, both stationary and mobile nodes are combined [10].
- *Coverage Property*: Based on spatial coverage of underwater sensor networks, localization schemes are classified into two-dimensional or three-dimensional. In the two-dimensional, all of the sensor nodes are assumed to be at the same depth on the ocean surface or the ocean bottom, or they may be floating at a certain depth. In the three-dimensional, each sensor node may be floating at various depths [10].
- *Location Computational Mechanism*: Based on the calculation technique of each sensor node location, two groups are defined: distributed and centralized. Distributed localization techniques allow each sensor to estimate its location individually. The localized sensor nodes do not know their locations, because the location of each sensor node will be calculated in a command center or sink.
- *Distance Measurement Method*: The localization schemes based on distance estimation method can be broadly classified into two categories: range-based or range-free. Precise distance or angle measurements made to estimate the location in range-based techniques, while range-free schemes do not use range or bearing information [4]. Instead, connectivity and hop-count metrics are employed to extract the node position.
- *Accuracy*: Regarding to the accuracy level of localization method, fine-grained and coarse-grained techniques are available. Fine-grained techniques are referred to that type of algorithms which infer the position of unknown nodes by manipulating ranging measurement techniques like TOA, TDOA, AOA or RSSI. These methods provide highly accurate location estimation. Instead, coarse-grain localization has lower accuracy while they do not employ any ranging metrics. This low accuracy may be sufficient for some applications like animal tracking.
- *Message Exchange Property*: In some localization schemes, only anchors are allowed to send beacon messages and the underwater sensor nodes passively listen to these localization signals. They are called silent nodes. Active localization allows underwater nodes to also participate in the localization procedure and send messages for localization. Active methods have higher communication overhead and higher energy consumption. But, silent protocols generally employ more number of anchor nodes with long-range communication capabilities [10].
- *Localized-nodes Cooperation*: based on the cooperation of localized nodes, two groups of schemes are definable: single-stage *vs.* multi-stage. In multi-stage, the localized sensor nodes act as new anchors and they are called reference nodes. Ordinary nodes do not become new reference nodes to help localize other ordinary nodes in single-stage schemes [9]. One of the drawbacks of multi-stage methods is error accumulation. Reference nodes propagate their locations as beacon signals while it may be estimated by errors in distance estimation and so on.
- *Anchor-node Deployment*: Localization techniques utilize anchors as location-aware nodes. These nodes and their deployments in the underwater networks have direct impact on localization process. Based on it, two different deployments are available: surface anchors or underwater anchors. On the one hand, to simplify the process of endowing these nodes with their positions, they are placed on the surface as GPS-enable buoys. Moreover, it is not always feasible to deploy anchor nodes at the sea floor, especially for deep ocean environments [14]. On the other hand, existing 3D underwater localization schemes require non-coplanar anchor nodes to uniquely localize a sensor, which implies that at least one anchor with position information needs to be underwater. The drawback of latest deployment is either infeasible or cost-prohibitive [15].

### 2.2. Localization Protocols Proposed for UWSNs

In this section, we briefly overview on investigated localization schemes for UWSNs. There are a couple of studies on localization for underwater acoustic networks. For example, underwater “GPS” systems [16] and PARADIGM [17] have been proposed based on surface buoys and one-hop communication [8].

Chandrasekhar *et al.* [18] proposed an Area-based Localization Scheme (ALS). In this method anchor nodes periodically send out beacon signals at varying levels. A two-dimensional region is partitioned into non-overlapping subareas by the ranges of the different levels of anchor nodes. Each non-localized underwater sensor node keeps a list of anchors and corresponding lowest power level and forwards this information with the sensor data to the sink. Sink is able to estimate the node's location. This scheme provides an estimation of a sensor's location within a certain area, rather than the exact location (range-free localization technique).

This method is a centralized method because the sink is responsible for estimating the node location where the ordinary sensor node resides [10,18]. ALS involves a high number of message exchanges and high energy consumption due to the sending of localization information to the sink [10], while it is able to estimate its location, already. Moreover, it is a stationary architecture network, so ordinary sensor nodes which are closer to the sink have lower lifetime rather than those further away from the sink because all messages must pass through them to reach the sink.

Centralized location computational mechanism in static networks with periodic beacon signals is less efficient compared to distributed ones, while non-localized sensors achieve localization information via periodic beacon signals, so they are able to estimate their location, already before sending information to the sink, but this computational mechanism saves sensor energy in terms of calculating the position.

A Dive and Rise Localization (DNRL) protocol is proposed by Erol *et al.* [19] for a mobile underwater wireless sensor network which utilizes mobile DNR beacons for localization. As GPS signals cannot propagate far through the water, the Dive ‘N’ Rise beacons carry GPS receivers and gain their coordinates when they rise to the surface and broadcast their positions, periodically in deep water to help underwater node's localization when they dive. Ordinary sensor nodes use TOA range measurement technique to calculate their distance to the DNR beacons.

The main idea behind TOA method is based on the simple known followed equation:

(1) x=v.t

where x is defined as distance between two sensor nodes and will be measured by using time propagated signal and signal velocity. With this technique, measured distance between two nodes i and j is derived by [11]:

(2) dist(i,j)=(t2−t1).v

where t1 and r2 are send and receive time, respectively.

The distance estimates and the coordinates of anchors are used in lateration. Lateration can be used to estimate *n* coordinates if there are *n* + *1* or more beacon messages [2]. DNRL is a 3-D distributed method with high energy efficiency and provides accurate estimates because the beacons update their locations, periodically and consider mobility of water currents, but this method requires a large number of DNR beacons for high localization success and they are more expensive beacons [10,19].

In [20], Multi-Stage Localization (MSL) is proposed to solve the coverage problem of DNRL by adding an iterative localization phase. In fact, it is the extended version of DNRL which dives to a predetermined depth. It is a multistage method which means the successfully localized nodes act as new anchor nodes and help others to estimate their locations, but the major drawback of this method is its high overhead due to iterative localization, so it is less energy efficient than DNRL. Moreover, the localized nodes provide their estimated locations with its estimation errors. Errors will be accumulated by the nodes which use the localized node's coordination as anchor node's position [10].

In [21], Three Dimensional Multi-power Area Localization Scheme (3D-MALS) is proposed which extends 2-DALS to three dimensional underwater networks. The proposed algorithm combines the idea of variable transmission power levels of ALS [18] and adds the vertical mobility of anchor nodes from DNRL method [19]. The network comprises four types of node, which are surface buoys, Detachable Elevator Transceivers (DETs), anchor nodes and ordinary sensor nodes. In [19] surface buoys are equipped with GPS on the water surface and a DET is attached to a surface buoy [21] and act as a DNR beacon. They broadcast their coordinates at different power levels. Non-localized nodes record mobile anchor location information and their respective lowest power level value and send these to the sink node to estimate the location [10]. It is also a centralized method similar to ALS and also introduces additional overhead by sending the related localization information to the sink where they can already estimate self-location using the periodic anchor messages [10].

A Silent Positioning Scheme is proposed in UPS model by Cheng *et al.* in [22]. This method is distributed and works for stationary underwater acoustic sensor networks (UASNs). Silent positioning is a localization scheme for one-hop underwater networks [22]. Four anchors are employed which sequentially send out beacon signals. The underwater nodes do not send any localization messages, hence UPS is silent. The overhead and energy consumption of UPS is low. It is a range-based method which utilizes TDOA range measurement technique, so it does not require synchronization, but UPS is only able to localize the nodes where they reside inside the area enclosed by four anchor nodes [10,22]. Moreover, the anchor locations need to be fixed and their positions require to be known by the sensor nodes which is not possible or hard to gain in practice [10]

USP is proposed in [15] for underwater localization in sparse 3D acoustic sensor networks. It is a projection-based localization method which is able to map the positions of the anchor nodes to the plane containing a to-be-localized sensor node to transform the problem of 3D underwater localization into a 2D positioning problem. The scheme is composed of two main phases: a predistribution phase and a distributed localization phase. The first phase repeats iteratively. In each iteration, each sensor performs a local broadcast of any new position information that it has. This causes high communication overhead and energy consumption [10,23].

AUV-Aided Localization (AAL) is proposed in [24] for a hybrid, three dimensional UASN. An Autonomous Underwater Vehicle (AUV) is utilized as a mobile beacon. Three different messages are used by the AAL method: wakeup, request and response messages. A wakeup message is broadcast by AUV to declare its presence in the communication range of the sensors. The wakeup message receiver sensor triggers a range measurement and sends a location request message. The response message will be sent by the AUV to answer the request message and it includes the AUV coordinates. The AUV receives GPS signals while floating. The number of necessary response messages differs with the localization algorithm used [24], such as triangulation or bounding-box [25]. This method is silent in terms of messaging properties [10], but it is obvious that they should send a request message to the mobile beacon (AUV), so sending activity is unavoidable in localization, even in silent methods. The accuracy of AAL will be improved by frequently location calibration, however this quickly exhausts the battery of the AUV. Another drawback is high localization delay due to the slow speed of AUVs [10].

Localization with directional beacon [26] is an anchor-free localization method which utilizes an AUV as mobile beacon like the AAL method. In LDB, the AUV uses a directional acoustic transceiver. It is a range-free scheme and an extended version of UDB [27] while it assumed node deployment in 2D and LDB works for three-dimensional underwater sensor networks. Sensor nodes listen to the broadcasting messages from the AUV silently and estimate their locations based on the location of the AUV at the time of entry and exit from the communication range of the sensors. LDB is not accurate enough because its accuracy suffers from both vertical and horizontal errors. Moreover, its accuracy depends on the frequency of AUV messages [10], although it is an energy-efficient localization method.

In [28], a multi-stage localization scheme is proposed for three dimensional underwater networks. 3DUL is divided into two different phases: a Ranging phase and a Projection and Dynamic Trilateration phase. It is a synchronization-free method, but involves high message exchange. Moreover, 3DUL has high localization delay that affects the localization accuracy in mobile underwater acoustic sensor networks [10].

Scalable Localization with Mobility Prediction (SLMP) is proposed in [8] by Zhou *et al.* It is a prediction-based localization method and it is assumed that every sensor node needs to determine its location periodically. Localization is performed in the same hierarchical architecture of LSL [29], with buoys, anchors and ordinary sensors. The localization process comprises two sub-processes: anchor node localization and ordinary node localization. First, the time is divided into multiple prediction windows with length set to *T_w_*. Window length *T_w_* should be an integer multiple of the localization period *T*_1_, which is the period every node needs to get its location. In every localization period, anchor nodes can easily communicate with the surface buoys, but the authors omit how the anchors localize themselves [2]. They could also predict their future mobility patterns based on their past measurements. At the same time, an anchor will calculate its estimated location and check the validity of the predicted location. If the error between the estimated location and the measured location is smaller than the stipulated threshold *S_t_*, then the anchor mobility model is valid. Otherwise, anchors should determine the new mobility pattern and broadcast it to ordinary sensor nodes. While in SLMP, anchor node localization is a challenge, itself. For ordinary node localization, each node maintains a reference list to record all its known reference nodes (nodes with known locations and confidence value higher than the confidence threshold). In a localization period, if a nonlocalized ordinary node does not receive any localization message, it will update its current location estimation using its previous location estimation and its predicted speed vector. If it receives a localization message from a reference node, it will update its reference list and perform new location estimation [8]. Communication overhead and energy consumption in prediction-based schemes such as SLMP depend on the mobility pattern [2]. Moreover, the length of localization period *T*_1_ has a reverse influence on the communication overhead. Other drawback of SLMP is its high computational complexity [30].

Related to different localization schemes and their performances, various literatures and surveys are available [4,9,10]. Overall, in localization of underwater wireless sensor networks, range-based schemes are better choice because range measurements using acoustics are much more accurate than when utilizing radio [31]. Range free methods have high communication overhead and energy consumption [10]. TOA-based ranging techniques are generally the preferred mode of range-based schemes [4], but require synchronization. If the UWSN is used for a long term mission, an additional synchronization protocol needs to be executed before localization [2]. Most of underwater applications demand distributed localization while they establish localization process with periodic beacon signals. Hence, ordinary sensor nodes have enough localization information to estimate their location on board. On the other hand, centralized localization computational methods preserve the energy of sensors.

### 2.3. Message Exchange Mechanism

A major constraint of underwater acoustic (UWA) networks is their limited energy supply [32]. Whereas the battery of a wireless modem can easily be recharge or replace on land-based systems, the replacement of an underwater modem battery involves ship time which is costly and time-consuming. Therefore, transmission energy is precious in underwater applications [33].

The energy consumption of acoustic modem is asymmetric, where the transmit power is often 100 times more expensive than that of the receiver mode [34], so acoustic transmissions are very power demanding. Moreover, the available bandwidth of underwater acoustic channels is severely limited. However, the receive mode in acoustic modems are inexpensive in terms of power consumption, but it has the same impact like transmit mode in limited bandwidth and increase the communication overhead. Consequently, in USN, localization protocols are expected to avoid excessive overhead and establish message exchange mechanism with the least exchange messages, both received and transmitted. In Table 2 we present a comparison between average communication cost in different highlighted and efficient existing underwater localization schemes which were described in Section 2.2. We illustrate the message exchange mechanisms of these methods based on receive and transmit messages from both anchors and ordinary sensor nodes.

It is obvious that some schemes in Table 2, like DNRL, UPS and LDB do not consume transmitting energy from ordinary sensor nodes. The silent positioning features of these methods can conserve bandwidth and improve network throughput since underwater sensors do not transmit any message for positioning purposes, but they have low average response times and they are only suitable for applications involving gathering of environmental data.

On the other hand, active localization methods involve intensive messages and also the localization response times are not fast enough for surveillance and prevention applications. Hence, RLS is the novel localization method with least possible message exchange and fast reaction to the events. RLS significantly decreases the number of messages transmitted by underwater sensor nodes. Moreover, the small packet size of RLS deserves further emphasis. None of the existing underwater localization schemes consider reducing the transmitted packet size in order to preserve the energy consumption and increase the available bandwidth. RLS considers disaster prevention and coastline protection applications like estimating tsunami location occurrence in deep waters to inform early warning alarms.

## 3. RLS Design

In this section, we present RLS, a Reverse Localization Scheme for underwater sensor networks. RLS is based on an event-driven location applicant message which minimizes the number of message exchange for localization. The scheme is composed of two main phases: a transmitting phase and a centralized geometric localization phase. During the first phase, ordinary sensor nodes observe and detect an event and immediately send a message toward the surface. The virtual position of detector sensor will be projected to water surface in order to solve 3D localization problem into 2D. While the latter executes the centralized localization in an onshore sink. Before presenting RLS, we describe its network model and underlying assumptions.

### 3.1. Network Model and Assumptions

**Figure 2.**
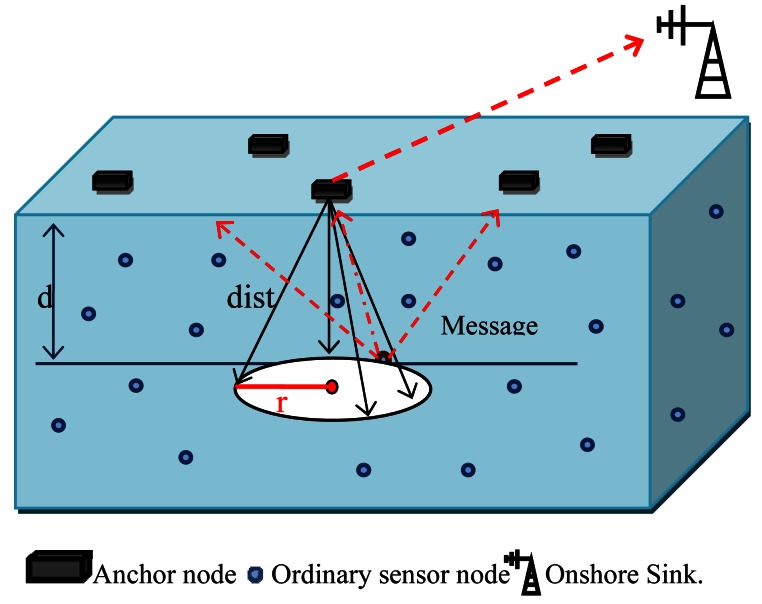
RLS architecture.

To accomplish a localization task in underwater wireless sensor networks, we consider three dimensional UWSNs where sensor nodes are randomly distributed in different depth of water. A possible deployment of 3-D underwater for RLS design is shown in Figure 2. Underwater nodes are assumed to be equipped with pressure sensors by which they learn their depth information. The network architecture consists of two different types of nodes and also a sink station to establish the centralized localization.

- *Ordinary sensor nodes*. Sensor nodes float and mobile with water currents at different depth in order to observe or detect a phenomenon. These are low-complexity sensor nodes which is equipped with pressure sensor to calculate depth information, so z-coordination is available in underwater environments and 3-D localization problem is transferred to 2-D localization. Underwater sensor nodes have limited power and should preserve their energy.
- *Surface anchor nodes*. Anchor or beacon nodes are location-aware to help ordinary sensor nodes to estimate their location. Since it may not be practical (either infeasible or cost-prohibitive) to place anchors on the sea floor in 3-D UWSNs [15], they are usually deployed at the surface like buoys and are equipped with GPS, RF and acoustic transceivers.
- *Sink or base station*. The base station is the data gatherer, which is usually a common computer or embedded system with higher process capacity [20]. The localization task is occurred in sink, as soon as it receives sensed data.

### 3.2. Transmitting Phase

This phase proposes a new message exchange mechanism which is based on *event-driven* reporting. All previous literatures trigger the localization process with broadcasting beacon signals, periodically. While these periodic messages are severely damaged due to the very low bandwidth of acoustic channels and highly increased overhead, the RLS scheme proposes a reverse transmitting phase to avoid excessive overhead and significantly reduce the number of messages exchanged

**Figure 3.**
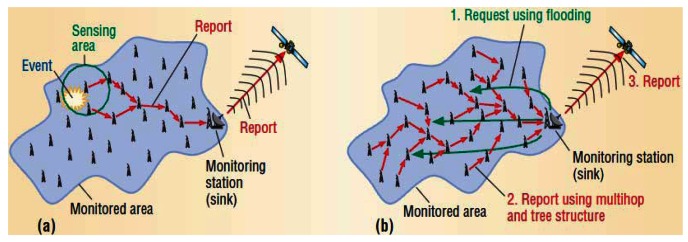
(**a**) Event-driven report; (**b**) Demand-driven report.

In terms of transmitting a message, two different types of reports are available such as *event-driven* or *demand-driven* [23]. In *event-driven* based transmitting, one or several sensor nodes detect an event and report it to a monitoring station, while, in *demand-driven* reporting sensors remain silent until they receive a request from the monitoring station. Figure 3 illustrates this concept more clearly.

RLS is a Reverse Localization Scheme, whereby localization is triggered from a/some detector sensor(s) in deep water and broadcast a localization applicant message toward the surface anchors. The first step in the transmitting phase starts whenever a phenomenon is observed or detected by an underwater sensor, it immediately broadcasts a message toward anchors on the surface using acoustic signals.

**Figure 4.**
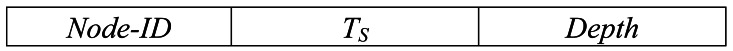
Localization applicant message format.

The signal reaches the surface anchors via the most direct path in order to reduce the delay and also decrease the number of message exchanged in the network. The multiple nondirect path signals will always arrive at the receiver antenna later than the direct path signal [22]. It is practical solution while most of acoustic modems have large range values because they are designed to work in applications where the distance between nodes are in kilometers [2], as addressed in Table 1. Moreover, the packet size of RLS is significantly reduced to transmit directly. The localization packet format is given in Figure 4, where *Node-ID* is the unique identification number of the message sender; *T_S_* (sending time) is the time when this message is sent and *Depth* is related *z* coordination of node location which is available from pressure sensor.

*T_S_* field is employed to estimate the distance between detector sensor and receiver anchor. The distance is measured with the Time of Arrival (TOA) technique, which will be described in Section 3.3. It is obvious that the size of the sent message is significantly reduced compared to existing localization algorithms such as ML, SLMP and ARTL [7,8]. In sensor networks, energy consumption is related with several parameters: a significant portion of energy is spent during packet transmission. Transmission is an unavoidable activity in both periodic and event-driven localization processes. Therefore, energy consumption is related with the number of transmitted bits [2]. As mentioned, the small size of packet in RLS reduces the energy consumption when an acoustic channel with low bandwidth is utilized.

**Figure 5.**
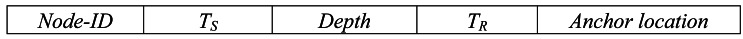
Completed message by anchors.

The second step in the transmitting phase starts as soon as the surface anchors receive the localization request message from detector sensor(s) in deep water. They insert T_R_ (receiving time of message) and also their location, where they equipped with GPS. After that, all receiver anchors forward completed message to the sink station. Figure 5 illustrates the completed localization request message which is forwarded by receiver anchors to the sink station.

These messages have some more data bits compared to the localization request message which is sent by sensors. Anchors utilize RF signals to forward the message to the sink through the air with high bandwidth and more energy.

### 3.3. Localization Phase

RLS is a centralized localization method where the sink is responsible for estimating the detector sensor location. The message completed by the anchor is sent to the sink station. The sink collects information from anchor nodes to estimate the node location. In order to save the energy of ordinary sensor nodes and anchor nodes and also to decrease the localization response time, location estimation occurs at the sink.

Suppose there are *M* surface anchors on the water surface, their *x-y-z* coordinates are (*X_i_, Y_i_*, 0) for *i* ∈ [1, *M*] and *S* numbers of ordinary sensor node where their coordinates are (*x_j_, y_j_, z_j_*) for *j* ∈ [1, *S*]. RLS establishes localization process from a sensor which is applicant for localization. Hence, the detector sensor does not have any anchor location information. Using a centralized localization is an advantage of RLS where detector sensors do not have to wait to receive beacon signals to estimate their positions. Based on the localization request message shown in Figure 4, an ordinary sensor only acquires depth information by using a pressure sensor where it is assumed that *z_j_* is extracted based on this field. Consequently, the 3-D localization problem becomes a 2-D position estimation problem [33]. Position estimation is based on the lateration method which is a widely used technique and is also employed by GPS systems. Lateration can be used to estimate *n* coordinates if there are *n* + 1 or more beacon messages. The method is based on the idea of intersecting circles [2] where the anchor is centered in the circle and the radius of the circle is the distance between the anchor and sensor. Let *dist* (*i, j*) be the distance between surface anchor *i* and detector sensor *j* and it will be calculated by sink based on *T_S_* and *T_R_* field via using TOA as soon as sink receives an anchor message shown in Figure 5. After receiving the anchor message in sink, *dist* (*i, j*) is defined as:

$$\text{dist}\left(i,j\right)\in \left\{s|s\in LS_{a}\right\},\;\text{where}\;LS_{a}=\pi r.\text{dist}.$$

*LS_a_* is Lateral Surface area of the cone shape made in Figure 2. In fact, all possible distances with the same calculated value by TOA at the unique *z* coordinate based on depth field is made the lateral surface area of this cone. In next step during localization phase, detector sensor coordination should be estimated by sink. Basically, the estimated coordinates should satisfy a set of equations:

(3) $${\left(x_{j}-X_{i}\right)}^{2}+{\left(y_{j}-Y_{i}\right)}^{2}+{\left(z_{j}-Z_{i}\right)}^{2}={\text{dist}}^{2}$$

As mentioned before, *Z_i_* = 0 and *z_j_* is available, so equation (3) is changed to (4) as follows:

(4) $${\left(x_{j}-X_{i}\right)}^{2}+{\left(y_{j}-Y_{i}\right)}^{2}={\text{dist}}^{2}-{z_{j}}^{2}$$

In Figure 2, it is obvious that *z_j_* is the height of the cone, so *z_j_* = d and the right side of Equation (4) is changed to the following expression:

(5) $${\left(x_{j}-X_{i}\right)}^{2}+{\left(y_{j}-Y_{i}\right)}^{2}={\text{dist}}^{2}-d^{2}$$

where *dist^2^* – *d*^2^ is equal with the radius of the circular base of the cone shape in Figure 2 and is shown by *r*. This value is extracted from the simple equation in (6):

(6) $$r=\sqrt{{\text{dist}}^{2}-d^{2}}$$

Value *r* is calculated by the sink and it inserts *r* into the localization message received from the anchor. Hereinafter, this packet will be recorded in the sink as a localization message. Based on this message, The position of an ordinary sensor is estimated as follows:

(7) $$\forall j\in S\to (x_{j,}y_{j})\in \left\{p|\sum p=\text{Circum Circular Base}\right\}$$

where *Circum Circular Base* is calculated from Equation (8):

(8) Circum Circular Base=2πr

To estimate the x-y coordinates of a detector sensor, according to lateration, only three localization messages are enough. When the sink reaches three localization messages and inserts the *r* value to them, the position of the node will be pertain to Equation (7) and intersects three circles according to lateration to achieve the actual node coordination. Hence, we could map the nodes from 3D to 2D with those determined circles. The RLS operation is outlined in Algorithm 1.

**Algorithm 1.** RLS operation.1: **if**
*sensor senses an event*
**then**2: *broadcast (localizationMSG) // including node-ID, T_S_*, depth3: **end if**4: **for** each *beacon receive (localizationMSG**) do***5: *localizationMSG.insert (T_R_, B(x, y))*6: *send LocalizationMSG toward sink*7: **end for**8: **for**
*sink receive (localizationMSG)*
**do**9: dist = (T_R_ - T_S_) * v10: *r = sqrt (dist*ˆ*2 - depth*ˆ*2)*11: *localizationMSG.insert(r)*12: *Sink.table.addrecord (LocalizationMSG)*13: *m = m + 1*14: **If *m*** ≥ *3*
**then**15: *Select 3 records which (r* < *others)*16: *P_S_* = *Trilaterate((x_1_,y_1_,r_1_), (x_2_,y_2_,r_2_), (x_3_,y_3_,r_3_))*17: **end if**18: **end for**

From Algorithm 1, it is seen that RLS is an event-driven method (line 1). In a sensor network, sensor nodes sample several properties from their surroundings, according to their deployment purpose. Temperature, pressure, salinity and acceleration are typical sensors for underwater sensor networks [2] where it is practical to use the proposed RLS algorithm. In RLS, the task of ordinary nodes is rather simple. They only broadcast a localization request message whenever they detect an event or determine the measured value (line 2).

The detector sensor does not require waiting to receive location information from beacon nodes. As for beacon nodes, the localization task is also energy-efficient and fast. They only receive the localization messages broadcast by sensors and insert their time and location in it (line 5). Then it is forwarded to the sink immediately (line 6). The transmitting phase of the RLS method finishes here (line 7).

The proposed message exchange mechanism can save energy by significantly reducing the number of sending packets. It does not involve intensive messages while only send an event which is desired value for network goal. At later time, if the position of node would be changed by water currents, the measured value is sent and location information is calculated in sink.

The localization phase will be launched by a localization message reaching a sink from a beacon (line 8). The distance between detector sensor and receiver beacon is calculated in the sink by using TOA (line 9). Then, the radius of circular base in Figure 2 will be estimated and the sink inserts this value in the localization message (lines 10, 11). Now, this updates message is recorded in a table in the sink to be used in estimating the location of the node (line 12). After collecting at least three messages from different beacons, trilateration is applied and the location of the occurred event is determined (line 16). Beacon signals with the lowest r value in the localization table are preferred to estimate more accurate locations (line 15), because these beacons are closer to the sensor compared to other beacons.

### 3.4. Mobility of Underwater Sensor Nodes

Underwater sensor nodes move continuously with water currents. Motion of underwater objects is related to many environment factors such as water currents and water temperature [35]. The water currents were modeled simply and also, their direction is considered. Their x and y coordinates change in the following way [19]:

(9) $$x\left(t\right)=x\left(t-1\right)+v_{cx}$$

(10) $$y\left(t\right)=y\left(t-1\right)+d_{t}.v_{cy}$$

where *d_t_* specifies the direction:

$$d_{t}=\left\{\begin{array}{c} -1\;\text{if}\;d\left(t-1\right)=1\;\text{and}\;y\left(t-1\right)>l_{cy}\\ 1\;\text{if}\;d\left(t-1\right)=-1\;\text{and}\;y\left(t-1\right)<-l_{cy} \end{array}\right\}$$

It is assumed that the main current is along the x-axis, with constant speed *v_cx_*, where *v_cx_* ∈ [0, *v_max_*]. On the y-axis, the nodes are allowed to oscillate by relatively small amounts (*l_cy_*) [19].

## 4. Theoretical Error Analysis

All measurement errors can be divided into two types: extrinsic and intrinsic. Extrinsic errors cover the physical effects on the measurement channel, such as multipath effects and changes in the signal propagation speed due to changes in the surrounding environment. On the other hand, intrinsic errors are caused by defects of hardware or software. Extrinsic errors are harder to handle or predict in realistic deployment, but intrinsic errors cause many complications. Position estimation is strictly influenced by even relatively small measurement errors [36]. Some extrinsic errors for our proposed localization scheme will be addressed in Section 5.3. In this section, we analyze RLS in detail and point out the possible localization errors which affect its accuracy.

### 4.1. Projection Accuracy

As described in the localization phase, the simple geometric relationship Equation (6) is employed to project the virtual location of detector sensors from deep water to the surface. The errors in the projection process can be estimated by the error propagation formula and are bounded by:

(11) $$\Delta r\leq \frac{\partial r}{\partial v}\Delta v+\frac{\partial r}{\partial T_{R}}\Delta T_{R}+\frac{\partial r}{\partial T_{S}}\Delta T_{S}+\frac{\partial r}{\partial d}\Delta d$$

where Δ*r*, Δ*v*, Δ*T_S_*, Δ*T_R_*, Δ*d* are the errors in *r, v, T_S_, T_R_* and *d*, respectively. Based on the equation (11), it is implied that projection accuracy for localization in RLS scheme directly depends on the errors in propagation delay, sound speed and estimated depth by presser sensors. Here, we analyze and address each in detail.

#### 4.1.1. Variable Speed of Sound

The speed of sound in water depends on the temperature, pressure and salinity. The speed of sound is addressed as an extrinsic error, as it depends on the surrounding environment and will be changed under various conditions. The underwater acoustic propagation speed is accurately modeled as [37]:

(12) $$q\left(z,S,t\right)=1449.05+45.7t-5.21t^{2}+0.23t^{3}+\left(1.333-0.126t+0.009t^{2}\right).\left(S-35\right)+163.z+0.18z^{2}$$

where *t* = T/10 (T is the temperature in °C), *S* is the salinity in *ppt* and *z* is the depth in km. the above equation provides a useful tool to calculate sound speed with an accuracy of 0.07 m/s, around 1,500 m/s. underwater sensor nodes can estimate the speed of sound using CTD sensors in advance, and this data is also stored during localization[28].

#### 4.1.2. Errors in Propagation Delay

Consider the model for the clocks of the sensor *S* and the anchor *A*:

$$f_{S}\left(t\right)=tf_{A}\left(t\right)=at+b$$

where *a* is the skew, *b* is the offset, and *t* is the global reference time. When the detector sensor node *S* and the anchor node *A* exchange timestamps, with *T_S_* = *f_S_*(*t_send_*), *T_R_* = *f_A_*(*t_send_* + *t_prop_*), the corresponding error in propagation delay can be calculated as:

(13) $$\Delta t_{\text{prop}}=t_{\text{prop}}-(T_{R}-T_{S})$$

#### 4.1.3. Errors in Depth Measurement

At an underwater environment, the depth information is usually obtained by measuring water pressure. The knowledge of the pressure-depth relationship measures the depth that is associated with the medium of interest. Even small amounts of error in depth measurement cause imperfections in the localization accuracy. Fortunately, current water pressure sensor technologies can provide very accurate underwater depth measurement [15,38]. Hence the effects of errors in depth measurement may cancel out.

## 5. Performance Evaluation

In this section, we evaluate the performance of our proposed localization scheme through extensive MATLAB simulations.

### 5.1. Simulation Setting

The parameters used for the related localization simulation are listed in Table 3. In our simulation experiments, we assume that underwater sensor nodes drift with water currents and they are able to self-organize in the network. The anchor nodes and the ordinary sensor nodes are distributed in a (1,000, 1,000, 600) volume [2]. The percentage of anchor nodes varies from 3%, 5%, 8%, to 12% in our simulation. The numbers of underwater sensor nodes vary from 100 to 500 [39]. They are also equipped with pressure sensors and acoustic modems. Most of the available acoustic modems have large range values because they are designed to work in applications where the distance between nodes are in the kilometer range [2], but long range modems achieve lower data rates. In RLS, we can successfully reduce significantly the number of transmitted bits compared with the existing localization methods like those described in [7,8,31]. Micro-modems are a special kind of underwater acoustic large range modem developed by the Woods Hole Oceanographic Institution [40], where it utilized by the RLS. Compared with the available modems such as Aquacomm modem developed by [32] with a range of 200 m, micro-modems have a large range of around 1,000 m [41]. Hence, they are suitable for applications in deep ocean water. We assume the nodes are synchronized and the distance can be measured by TOA [2]. We give the average of 100 simulation runs. Performance of the proposed localization scheme is analyzed in terms of localization success, localization accuracy, energy consumption and localization response time.

Localization success is defined as the ratio of the localizable nodes to the total nodes. Mean error ratio presents the localization accuracy and it is the average distance between the estimated position and the real coordinates of all nodes. As in [45,46], for our simulations we normalize this absolute localization error to the node communication range R [31]. Energy consumption is defined as spent energy for transmitting and receiving messages by both anchors and sensors divided by the number of localized nodes. Another useful metric is average response time for localization of a detector sensor. It is expected a small amount for localization schemes considering to the application which require very fast reaction.

**Figure 6.**
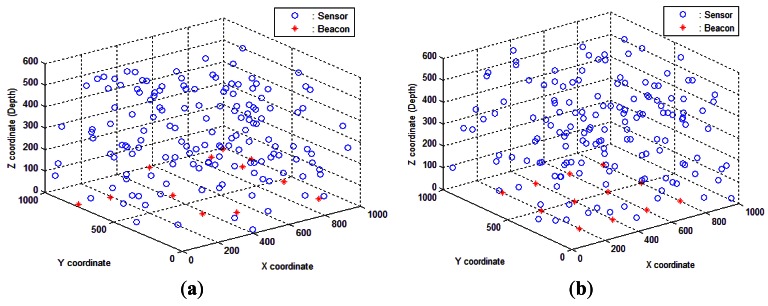
(**a**) Random distribution; (**b**) Grid anchor deployment.

Figure 6(a) shows the initial random sensor and anchor distribution, Figure 6(b) illustrates the grid anchor deployment and random sensor distribution.

### 5.2. Localization Success

Localization success is defined as the ratio of nodes in the entire network that could be localized successfully [2,28]. RLS is an event-driven localization scheme, so the ratio of localized nodes should be evaluated as detector sensors (the sensors that receive an event). In simulation, all sensor nodes will detect the event, at least once. The average localization success is defined as:

(11) $${\text{Avg}}_{LS}=\frac{\sum_{i=0}^{n}\frac{N_{l}\times N_{t}}{N_{d}}}{n}$$

where *Avg_LS_* shows the average localization success and *N_t_* is the total number of sensor nodes deployed in the 3D space. The number of localized nodes and the number of detector sensors are shown by *N_l_* and *N_d_*, respectively. We average the sensor location estimation over 100 trials, so *n* is the number of instances, which set to 100.

**Figure 7.**
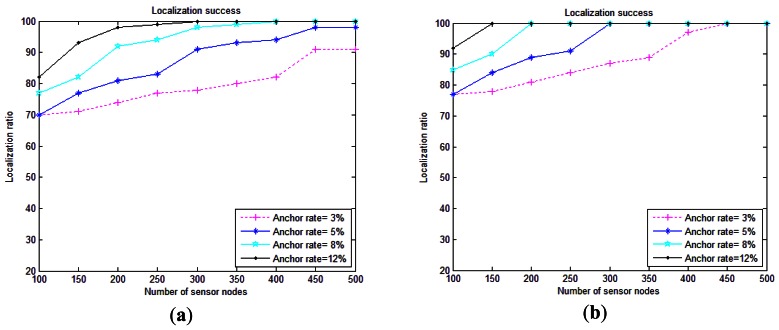
Average localization success. (**a**) Beacon nodes with random distribution; (**b**) Versus Grid deployment in water surface.

High average localization success is desired by RLS due to its targets for surveillance applications and disaster preventions. Consequently, a detector sensor is a should-do-localized sensor node in RLS localization scheme. This metric is illustrated in Figure 7.

High average localization success is desired by RLS due to its targets for surveillance applications and disaster preventions. Consequently, a detector sensor is a should-do-localized sensor node in RLS localization scheme. This metric is illustrated in Figure 7.

As can be seen, the localization success of RLS increases with increasing percentage of anchor nodes, while the ratio of localized nodes does not change much when we deploy more sensor nodes in the volume. Compared to SLMP [8], RLS can achieve a high level of localization ratio (approximately near 100%) with only 8% anchor nodes, while SLMP strictly depends on the node density to achieve around 95% of coverage with 10% anchor nodes [8]. It is shown in [47,48,49] that range-based distributed localization schemes have relatively high requirements on the node density of the network. This represents an advantage of RLS which is independent of the node density. Our proposed method can achieve a high localization ratio, as it is shown in Figure 7(a), without any requirement on the node density. The reason is that our scheme does not depend on communication between sensor nodes. It depends on communication with surface anchors. It means that this method is appropriate for both dense and sparse deployments. Figure 7(a) shows us that the localization ratio increases slightly with the number of sensor nodes, which is the result of the increase in anchors based on the node number percentage. Hence, we can infer that the more the anchor percentages, the higher the localization success. As illustrated in Figure 7(b), anchors with grid deployment help provide a higher localization ratio compared to random anchor deployment seen in Figure 7(a). The issue of anchor node's placement should not be neglected [30]. Anchor node's effective placement can greatly improve the localization ratio. In grid deployment, the localization ratio can reach to its highest level with 100% localization success with 150 sensor nodes and an anchor rate 12%, while in anchor random deployment, we should increase the number of sensor nodes up to 300 and the anchor rate to 12% to achieve the same amount of localization ratio. The reason is that in the anchor node deployment in grid manner, many more help transmit the messages to the anchors. In fact, this deployment guarantees the presence of at least three anchor nodes in communication range of each ordinary sensor node. Hence, the localization ratio will be increased. It is obvious that an anchor node's effective placement cannot use some localization schemes like SLMP [8], where anchors are floated among ordinary sensor nodes in the network.

Based on th comparison between RLS and LSL and DNRL in [2] with the same assumptions, it is obvious that our scheme outperforms both LSL and DNRL in terms of localization ratio. To reach around 80% of localization ratio, DNRL and LSL with 250 sensor nodes, require 10% beacon coverage. In contrast, RLS with only 5% of anchor ratio and the same node number can achieve this same level of localization ratio. Moreover, under the same grid deployment conditions, our scheme to reach 80% of localization ratio needs only 3% anchor ratio. For localization success of all 250 sensor nodes in DNRL and LSL, 35 beacon percentage is required, while in RLS the anchor ratios required are 12% and 8% in random and grid anchor deployments, respectively. DNRL and LSL pose high cost related with the high number of anchor nodes needed to achieve a proper localization ratio.

### 5.3. Localization Accuracy

The underwater environment presents unique challenges to accurate localization [15]. Accuracy is defined by the mean error ratio, which is the average of the difference between the estimated location and the actual location of a node for location estimation [2]. RLS is a projection-based localization method while the location of detector sensors should be projected by receiver anchors on the water surface.

There are some major sources of error in the localization process. By assuming the positions of anchor nodes are accurate enough, we can focus on the error sources between beacons and ordinary nodes. The localization error may arise from three sources [50]:

- Time stamping uncertainty: it is expected that the timestamp field is generated just before the sending and as soon as the packet is received, but some delays may affect the accuracy of calculated range based on timestamps [51].
- Projection accuracy: in other existing projection-based localization schemes like USP [15], the position of anchor nodes are transferred by ordinary sensor nodes in the plane of node residence in the depth of the ocean, while this occurs with anchors in RLS. Each receiver anchor node employs the simple geometric relationship based on Equation (6) to project the location of detector sensor into the water surface. It is obvious that both ranging errors and depth errors negatively affect the accuracy of RLS as far as projection accuracy is concerned.
- Underwater multipath fading: any underwater location detection system will be greatly influenced by underwater multipath channel fading [22]. Some major factors influencing underwater multipath fading include water temperature and clarity, motion behavior of receiver and underwater objects, and transmission range.
- Node Movement: some external factors, such as temperature variations, wind, and ocean currents and so on, may cause underwater objects to move slowly [44]. The influence of this motion on localization introduces some errors due to the continuously changing position during message exchanges. Fortunately, current underwater sensor nodes can control passive mobility well and limit the relative speeds between sensor nodes to less than 0.1 m/s [52].

**Figure 8.**
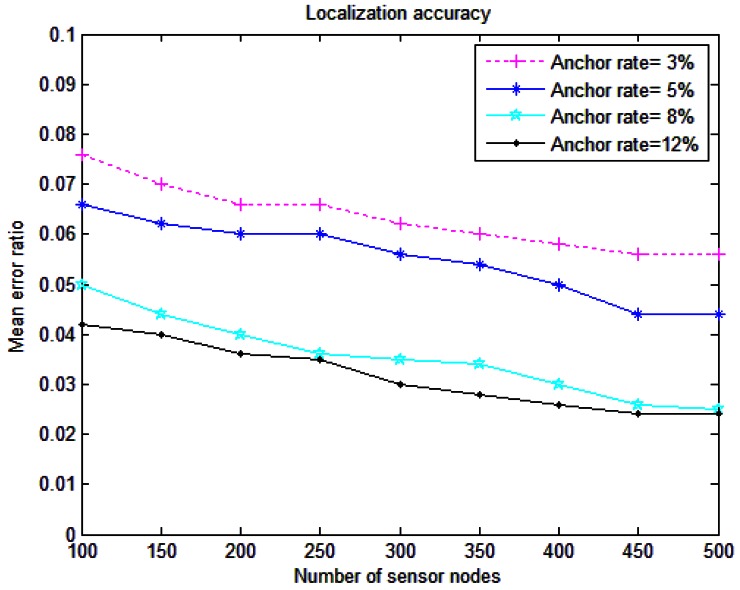
Mean error ratio for RLS.

Figure 8 illustrates the relationship between the mean error ratio and the number of sensor nodes. It is observed that with the increase of the nodes number, the accuracy of our scheme increases, but this increment is very limited, so the errors in RLS do not depend much on the node density. RLS works accurately for both sparse and dense underwater networks. Figure 8 also shows that the mean error ratio will decrease observably with the anchor ratio. For example, with 250 sensor nodes, when the anchor percentage is 3%, the localization error is around 0.07R. But when the anchor percentage is enlarged to 12%, it reduces to approximately 0.03. Thus, more anchor nodes can translate into smaller mean error ratios. This is because with the increase of anchor ratio, more anchors are available to receive the localization request messages from detector sensors, so the nodes have more anchors in their communication range to choose from.

### 5.4. Energy Consumption

In underwater acoustic channels the energy costs of acoustic modems are as: *Tx* > *Rx* > *Idle* ≫ *Sleep*, so a significant portion of energy is spent during packet transmission [2]. In contrast, energy costs for terrestrial radio modems are: *Tx* ∼ *Rx* ∼ *Idle* ≫ *Sleep* [53]. Therefore, in USN, localization protocols are expected to avoid excessive overhead and establish localization with the least possible number of messages. Moreover, for a mobile UWSN, localization should be repeated periodically which also increases the energy consumption and decreases the network lifetime. Hence, to calculate the energy consumption, we use the average number of received and transmitted bits and energy per bit values of a Micro-modem.

**Figure 9.**
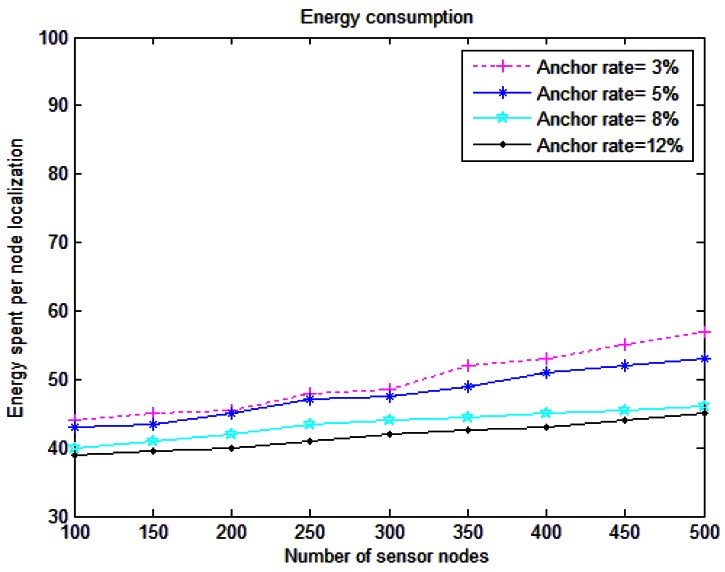
Energy consumption per node for localization.

Figure 9 shows the energy consumption for RLS. It is obvious that this value is increased by increasing the number of sensor nodes. Energy spent per node localization is defined as the total amount of energy consumption including transmit and receive for both anchors and sensors. RLS anchor nodes consume low amounts of acoustic modem receive power and low RF transmit power, so the energy consumption for anchors is very low and increasing the percentage of anchors parallel with enlarging the ordinary nodes, decreases the energy spent per node for localization.

In RLS, according to Figure 4, the number of transmitted bits by detector sensor is equal to 80 bps, so the energy consumption for sending and receiving one packet is calculated based on [50]:

(13) $$E_{Tx}=P_{Tx}\times \frac{\text{packet}-\text{size}}{\text{data}-\text{rate}}$$

(14) $$E_{Rx}=P_{Rx}\times \frac{\text{packet}-\text{size}}{\text{data}-\text{rate}}$$

where *E_Tx_* and *E_Rx_* are the energy consumption for sending and receiving one packet, respectively. Transmitted and received power are shown by *P_Tx_* and *P_Rx_*, respectively. Energy spent per node localization is defined as the total amount of energy consumption including transmit and receive for both anchors and sensors, so we can formulate it as follows:

(15) $${\text{Total}}_{\text{Energy–Consumption}}=\frac{E_{TA}+E_{TS}+E_{RA}+E_{RS}}{\text{localized node}-\text{num}}$$

where *E_TA_* and *E_TS_* are the energy consumption for transmitting by anchor and sensor, respectively. Energy consumption for receiving a message for anchor and sensor is shown by *E_RA_* and *E_RS_*, respectively.

It is extracted from Equation (15) that a significant portion of energy is spent during transmission by ordinary sensors. Anchor's transmission in RLS occurs by RF signals with very low energy consumption and it is negligible, so to calculate the energy consumption, we use the number of transmitted bits. It is obvious from Equation (13) that energy consumption will be reduced by decreasing the transmitted packet size. Our proposed scheme can reduce the energy consumption of localization in comparison with energy efficient methods like SLMP [8], which is a prediction-based scheme, via decreasing the message size. In SLMP [8], the localization message structure has some extra fields such as Location and Speed vector compared to the RLS message format (Figure 4). Moreover, this localization message could be sent by both anchor nodes and localized ordinary nodes [8]. As another comparison for energy consumption of RLS and ARTL In [50], the packet size and the data rate for ARTL are 400 bits and 1,000 bps, respectively. Like RLS, this method also utilizes the Micro-modem [44]. It is obvious that RLS with an event-driven report decrease significantly the message size and the transmitted data bits. The calculated *E_Tx_* in ARTL is 14 J. It is calculated that *E_Tx_* for our method based on Table 2 is around 35 J, but for data rates higher than 80 bps Micro-modems require additional coprocessor data to be recorded [44], so the ARTL method employing the Micro-modem can achieve lower energy consumption than our method, but it requires an additional coprocessor which adds extra cost and complexity.

Compared to existing energy efficient methods such as methods discussed in [2], the RLS scheme is much more energy efficient as they only involve the energy spent for estimating the location and omit the big proportion of energy consumption for sending the estimated location to the sink or beacons. In a real time localization method, the estimated coordinates will attach to the packet when the localized sensor reports a phenomenon, so the sensed data which is tagged with location information should be delivered to the sink or beacons for processing to finish the localization task successfully. RLS combines and merges all steps, from detecting an event to estimating the location in the sink and can achieve lower energy consumption by decreasing the number of messages exchanged. Its centralized computational mechanism helps the RLS improve energy efficiency.

As mentioned before, one of the practical solutions to reduce the energy consumption is by reducing the size of messages. As another solution, an energy efficient scheme should reduce the number of messages exchanged for localization process in order to localize an unknown node. Due to the low bandwidth, low data rate, high delay and high bit error rate the USN localization protocols are expected to avoid excessive overhead and establish localization with the least possible number of messages. In a large-scale USN, the messaging intensification of localization protocols may increase. In addition, for a mobile USN, localization should be repeated periodically which also increases the overhead. Considering all these challenges, it is essential to develop a localization scheme with low message exchanges. The number of messages sent or received by anchors and sensors are investigated for some highlighted underwater localization methods in Table 2. Based on this table, in order to exhibit the effectiveness of proposed approach, the exact number of exchange messages for localizing an unknown node is extracted and illustrated in Table 3. This table analyzes the communication cost for various localization methods. Communication cost is the number of messages sent by anchors and sensor nodes in order to localize an unknown node [7].

The reported number of messages is calculated under ideal conditions. In fact, these are the least message numbers required for localization per unknown node by various methods. For example, SLMP requires two messages when we assume that its prediction algorithm is valid. Otherwise it needs at least three messages. The highest message number for these methods can be extracted by Table 2. As it is implied from Table 3, RLS can achieve the lowest message exchange among acoustic channels compared to other methods. It even has less message number than silent schemes like D′N′R, UPS and LDB. In silent methods, unknown sensors do not transmit any message, but in RLS only one message for localizing one sensor node is sent by the sensor.

### 5.5. Average Response Time

**Figure 10.**
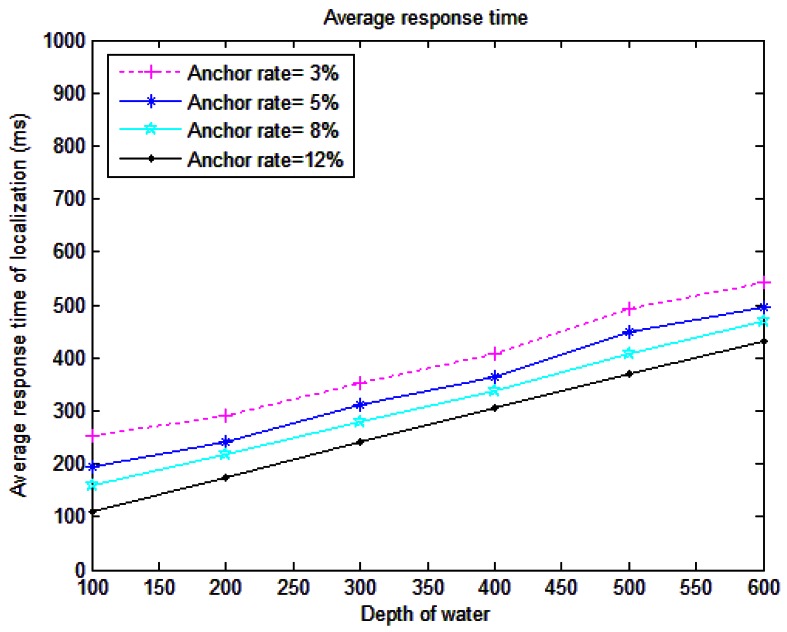
Average response time of localization.

RLS is considered for applications in which the average response time is a vital metric, such as for disaster prevention like tsunamis [54]. The period T is defined as the average response time where it is started from the time the event is detected by a sensor to estimating the location of the same sensor in the sink. In fact, the average response time is the average time to get a location estimate for the first time. Localization can be updated and refined in time but we consider the delay in estimating the location for the first time [7]. Figure 10 illustrates the average response time terms to different depths of water. In this simulation metric, we set the number of sensor nodes to 250. The localization time will increase with increasing depth of water. It is a logical relation, because the propagation delay of sound is high and will be increased by increasing the distance, so this is due to propagation delays. Higher anchor rates reduce the average response time because the number of anchors in communication range of detector sensor will be increased and they help to accomplish localization fast. Unlike all existing localization methods such as [2], RLS does not need to wait to receive anchor location information. It broadcasts its data, as soon as it senses something, so if the mobility of water current changes the node location, its position information is available in the sink to estimate its coordinates in real time when detecting the event.

Average response time is a vital component for estimating the performance of localization. In some applications like for an effective tsunami warning system, time is of the essence, while, it is not so crucial for ocean sampling applications. The time constraint for a tsunami early warning system is specified as [54]:

(16) $$T_{1}+T_{2}+T_{3}\leq T_{4}$$

where *T*_1_ is detection time, *T*_2_ is assessment time, *T*_3_ is evacuation time, and *T*_4_ is tsunami travel time. It is clear that to make the warning system effective, the time needed for detection should be minimized. On the other hand, most of the localization methods cannot reduce the detection time, because they must wait to receive the beacon messages. RLS is considered to reduce detection time and also reduce the response time via triggering of the localization process by the detector sensor. Delays in the RLS scheme only include the propagation delay and it is increased by increasing the communication range.

## 6. Effectiveness and Deficiencies of RLS

Our proposed localization scheme has the following deficiencies:

- RLS requires synchronization due to one-way ranging in TOA calculation like other existing range-based methods which utilize TOA such as, LSHL [31], DNR [19] and SLMP [8]. For time synchronization, more message exchange is required and higher energy is consumed. The nodes may be assumed to be synchronized for several weeks after initial deployment.
- We need to carefully plan the anchor node placement or increase the number of surface anchors to ensure that all nodes are always able to reach at least three anchor nodes in their communication range to avoid packet loss.

Some of effectiveness of the proposed approach is address as follows:

- It is an event-based localization method which can match duty-cycle environments. There is little research on UWSNs' localization problem for duty-cycle environment [30]. Sensor nodes can go to sleep according to some sleep scheduling mechanism. After detecting a phenomenon they can wake-up and launch localization tasks, so it is not required that all sensor nodes wake-up all the time.
- We can significantly reduce the packet size compared to other methods like ML [7] (96 bit), ARTL [50] (400 bit), and efficient localization method [31] (16 byte). Moreover, the number of messages exchanged is decreased by establishing an event-driven reverse localization where ordinary nodes do not have to wait to receive anchors' location information. The localization process is launched by detector sensors deployed at various water depths.

## 7. Conclusions

We have presented an event-driven Reverse Localization scheme (RLS) that can achieve high average response time in underwater sensor networks. This algorithm requires very low computational complexity and energy consumption. We have developed an accurate ranging scheme using TOA to determine the distance between beacons and ordinary sensor nodes. It has been shown via simulation results that the proposed localization algorithm involves low message exchange and low data rates with less average response time where localization time is a vital component in some applications of underwater networks like disaster prevention. As future work, we plan to improve the localization coverage with assuming a constant velocity for underwater sensor nodes except for the mobility of water currents. Moreover, we are going to propose a range-based synchronization-free method to improve the accuracy of RLS that does not require additional messages for synchronization.

## Figures and Tables

**Table 1.** Data rates and typical bandwidth for underwater channel with various ranges.

**Span**	**Range (Km)**	**Data Rate**	**Bandwidth (KHz)**
**Short range**	<1	∼20 kbps	20–50
**Medium range**	1–10	∼10 kbps	∼10
**Long range**	10–100	∼1 kbps	2–5
**Basin scale**	3000	∼10 bps	<1

**Table 2.** Message Exchange Mechanism in Terms of Communication Cost.

**Method**	**Anchor**	**Sensor**
**D′N′R**	Send = I × A	Send = 0
	Receive = 0	$\text{Receive}=\sum_{i=1}^{I}\sum_{j=1}^{A}Ca_{\text{j}}$
**MSL**	Send = I × A	$\text{Send}=\sum_{i=1}^{I-1}L_{\text{i}}$
	Receive = 0	$\text{Receive}=\sum_{i=1}^{I}\sum_{j=1}^{m}L_{\text{i}}{\text{Cs}}_{\text{j}}$
**UPS**	Send = A × I	Send = 0
	Receive = (2A − 2) × I	Receive = A × I
**AAL**	AUV-Send = 2I	$\text{Send}=\sum_{i=1}^{I}Ca_{\text{i}}$
	$\text{AUV‐Receive}=\sum_{i=1}^{I}Ca_{\text{i}}$	$\text{Receive}=2\sum_{i=1}^{I}Ca_{\text{i}}$
**LDB**	AUV-Send = I	Send = 0
	AUV-Receive = 0	Receive = (2r / v × T_s_) × Ca
**3DUL**	Send = 2I × A	$\text{Send}=\sum_{i=1}^{I}Ca_{\text{i}}$
	$\text{Receive}=\sum_{i=1}^{I}\sum_{j=1}^{A}Ca_{\text{j}}$	$\text{Receive}=2\sum_{i=1}^{I}\sum_{j=1}^{A}Ca_{\text{j}}$
**SLMP**	$\text{Send}=\sum_{i=1}^{k}A_{i(\text{not valid})}$	$\text{Send}=\sum_{i=1}^{k}L_{i}$
	$\text{Receive}=\sum_{i=1}^{k}A_{i}$	$\text{Receive}=\sum_{i=1}^{k}ca_{i(\text{not valid})}+\sum_{i=1}^{k}c_{s}$
**RLS**	$\text{Send}=\sum_{i=1}^{e}C_{\text{i}}$	$\text{Send}=\sum_{i=1}^{e}Sd_{\text{i}}$
	$\text{Receive}=\sum_{i=1}^{e}C_{\text{i}}$	Receive = 0

**I:** The total number of intervals. It is calculated based on $\frac{T}{T_{S}}$ where T is the localization period and *T_S_* is the time slice. **I,** will be decreased with increasing *T_S_*; **K:** The number of localization period; ***S_d_*:** The number of detector sensor; ***Ca*:** The number of sensor nodes located in anchor communication range. *Ca* is increased by enlarging the anchor communication range; *C_a_*_(_*_not valid_*_)_: The number of sensor nodes located in anchor communication range with invalid prediction pattern; ***L_i_*:** The number of localized nodes in *i^th^* interval; ***Cs*:** The number of nodes located in the communication range of the localized sensor nodes; ***A*:** The number of anchor nodes; ***A_not valid_*:** The number of anchor nodes which their mobility prediction is not valid; ***r*:** Sensor nodes communication range; ***v*:** The speed of AUV movement; ***C*:** The number of anchors located in ordinary sensor nodes communication range; ***e*:** The number of events which occurred in T.

**Table 3.** Simulation Parameters.

Parameter	Value
Localization Domain Area	1,000 m × 1,000 m
Maximum Depth	600 m
Beacon Percentage	3%–5%–8%–12%
Ordinary Node Number	100–500
Speed Of Sound	1500 m/s
Error in Speed of Sound	0.07 m/s [42]
Error in Depth	0.1 m
Standard Derivation Of Time stamping	15 μs [43]
Packet Size	80 bits
Data Rate	80 bps
Transmit Power	35 W [44]
Receive Power	0.3 W [44]
Simulation Run	100

**Table 3.** Communication cost.

**Method**	**Number of Messages**	**Description**
D′N′R	3	Anchor
MSL	3–6	Anchor(3) + Reference node(3)
UPS	4	Master-Anchor(1) + Anchor(3)
AAL	9	Anchor-weak up(3) + Sensor-Req(3) + Anchor(3)
LDB	2	AUV arrive to range + AUV exit from range
3DUL	7	Anchor-Req(3) + Sensor-Ack(1) + Anchor(3)
SLMP	2–3	Buoy + Referenced node + (Anchor)
RLS	1	Applicant Sensor
